# Sketch of the Proceedings of the National Medical Convention

**Published:** 1847-06

**Authors:** 

**Affiliations:** Philadelphia


ART. 4.—*Sketch of the Proceedings of the National Medical Convention. Letter from the Editor.*

PHILADELPHIA, May, 4th, 1847.

MY DEAR H:—

As our readers will be desirious of having some account of the doings of the convention in the June No. of the Journal, and the official proceedings will probably not be published in season to be rendered available for that No, I purpose, agreably to our arrangement, to send you a few diurnal *jottings*, endeavoring to embody the more important events which transpire during the session, with some notice of incidental matters pertaining to medical affairs in Philadelphia, which may appear to possess interest or novelty. My time will be so constantly occupied that what I write will necessarily be scribbled very hastily, and will doubtless need the fullest indulgence that our readers can bestow, alike for faults of omission and commission.

We arrived yesterday at 2½ P. M., and proceeded, at once, to report at the Academy of Natural Sciences, the place selected for the meeting of the Convention. We found that several delegates had preceded us. We registered our names, and received a printed ticket which serves as a card of admission to the Hospitals, Colleges, and various other public institutions of the city. The committee of arrangements appointed by the delegates from Philadelphia were in attendance, and welcomed us with great cordiality.

A word or two respecting the place of meeting. The Academy of Natural Sciences is an association for the promotion of objects connected chiefly with Natural History. Their edifice embraces a lecture room, library, and museum—the latter constituting much the greater portion of the building. The Library is said to be the best in the country relating to subjects of Natural History. Their Museum is probably by far the most complete and valuable. The ornithological collection is especially extensive, and is perhaps the largest in any country. The department of comparative anatomy presents a goodly number of preparations. I observed some very large and valuable fossil remains. But not the least interesting was the cabinet of skulls collected by Dr. Morton, numbering nearly a thousand specimens, and embracing heads of all nations, ages and historical epochs. Upon an analysis of this vast collection Dr. Morton bases the classification and deductions contained in his splendid work. The Academy of Natural Science was originated by young men, and has received munificent contributions from some of the opulent friends of the objects for which it was instituted. Among the latter class is a present member, Dr. Wilson, who has enlarged the edifice entirely at his own expense, contributing some ten thousand dollars for that purpose, besides depositing the ornithological collection, and making other additions to the museum of various rare and costly specimens. The fossils, already referred to, have been lately contributed by Dr. Wilson. How wise and judicious, as well as generous, appears this method of investing a portion of superabundant wealth, when contrasted with that miserly hoarding which only contributes to the anxiety of the possessor, and perhaps inflicts upon heirs the greatest misfortune which could befal them! What glorious results for science, and for humanity, were attainable, if they who have been prospered in the accumulation of riches more generally appreciated, not only the benefits which they are able to confer upon society by liberal contributions to scientific objects, but the honor and satisfaction which they might therefrom derive!

Through the politeness of some of our friends, Dr. B. and myself attended last evening one of the medical clubs of the city. Several associations under this title exists here. The one at which we were present numbers some twenty or thirty members, meetings being held weekly at their houses in rotation. The meetings are entirely informal, every one coming and going, without ceremony, according to their conveinence or inclination. Medical subjects are not discussed except as matters of conversation. A simple entertainment is provided, and the meetings are, in fact, chiefly for the cultivation of social intercourse. We passed a delightful evening, meeting several, whom we had long known from their professional writings, and with whom it was a great satisfaction to form personal acquaintance.

If there be any one means of which our profession is to be especially elevated and improved, in my humble opinion, it is by cultivating among ourselves friendly relations; but as I have written on former occasions so much on this topic, I will not amplify upon it now. Let physicians know each other, look upon each other as brethren, and cultivate habits of amity and social intercourse, and there will be less, if not little need of codes of medical ethics, or of national conventions to reform abuses and elevate the character of the profession.

We visited (I use the plural *we*, not in an editorial sense, but as embracing my worthy coleague Dr. B., and myself) this forenoon by the polite invitation of Dr. Fox, the surgeon now on duty, the institution known as *Wills' Hospital*. This is a charity for indigent blind and lame, and owes its origin to the bequest of the entire property of James Wills, a citizen of Philadelphia. The original sum bequeathed was about one hundred thousand dollars of which about one half was expended in the purchase of the ground, and the erection of the present edifice. The portion unexpended yields an income of somewhat more than three thousand dollars, which goes to sustain the institution. It has, however, received aid from other individuals. The fund was placed by the testator under the control of the city authorities, who are the managers of the Hospital. It went into operation in 1832. The building is a neat structure of red granite, 80 feet front by 50 deep, and will accommodate about 70 patients. Four Physicians, and the same number of Surgeons, are annually appointed, who render their services gratuitously. The care of the patients devolves chiefly upon the surgeons. Nearly all now in the institution, amounting to forty or fifty, are cases of ophthalmic diseases, illustrating some of the most interesting of the varieties and different phases of these diseases. Cleanliness and good order (two cardinal points in the successful management of all such charities) are, apparently, fully carried out at this institution. The names of the surgical officers are sufficient guarantees that patients admitted there receive the advantages of the most approved principles of treatment skillfully applied..

*Wednesday, 3rd.*—At the hour appointed for the meeting of the convention, (10 A. M.) a large concourse of members and spectators had assembled, and I trust the remark is excusable, (as these notes will meet the eyes of some unprofessional readers) that my first sentiment was one of admiration of the *ensemble* which the convention presented. In respectability and dignity of aspect, and the indications of intellectual and moral qualities of superior order which are delineated in physiognomy, this convention will not suffer by comparison with any other public body. Said Prof. M., who sat near me ,"I have been a poor doctor all my life, and never realized 'till now the high respectability of the body of which I am a member." I fervently hope that the proceedings of the convention will not in any measure fail to confirm there first impressions.

The meeting was called to order by Dr. Hays, who moved that Dr. J. Knight, of New Haven, the president of the last convention, take the chair, and the former secretaries resume their seats until permanent officers are elected. The motion was carried, and at once acted upon. A committe of five were appointed to receive credentials, and report the names of accredited delegates. This committee shortly reported that all but six states of the Union were represented. The states not represented are, Maine, North Carolina, Iowa, Florida, Texas and Alabama.

The election of officers next came up, the convention deciding to elect a president, four vice presidents and three secretaries. A nominating committee, consisting of one delegate from each state represented, was appointed by the chair to present suitable names for these offices. After a short conference the following persons were selected, and unanimously chosen:—

***President.***—Dr. Knight, of Connecticut.

***Vice Presidents.***—Drs. A. H. Stevens, of New York; Wood, of Philadelphia; Carpenter, of Louisiana, and Buchanan, of Ohio.

***Secretaries.***—Drs. Stille of Philadelphia; Arnold of Georgia, and F. Campbell Stewart, of New York.

Various resolutions were submitted respecting inviting and admitting members of the Profession to participate in the convenvention who were not present as delegates. They were all negatived with exception of an invitation to medical officers of the Army and Navy.

The convention next proceeded to hear the reports of the several committees appointed at the previous convention, taking them up in the order in which they were appointed.

The first was of the committee appointed to report a plan of organization for a *National Medical Association*, Dr. Watson, of N. Y., Chairman. The report having been read, was ordered to lie on the table, in order to be printed for the use of the members. The same action was had on the other reports. I will defer any account of their features, recommendations, &c., until the convention takes action upon them. The committee (Dr. Griscom chairman) to consider the expediency and mode of procuring the registration of deaths, births and Marriages by the several State Governments, next reported an address which was submitted in order to be printed, and (if accepted) extensively circulated. The convention now adjourned to meet at 7, P. M. At the evening session, after receiving letters inviting the convention to visit the house of refuge, and the deaf and dumb asylum, the reception of reports from the committees was resumed.

Dr. Haxall, chairman of committe on an uniform standard of requirements for a degree, presented a report.

The committee on preliminary education, reported by their chairman, Dr. Couper.

Dr. Bell, chairman of committee, submitted a code of medical ethics.

The convention then adjourned to assemble at 9 A. M., to-morrow.

*Thursday*, *6th*.—Convention assembled at 9 A. M. The names of several new delegates were reported, the whole number now present amounting to two hundred and twenty-nine. Invitations were received to visit the institution for the education of the blind; and from members of the Medical Faculty of Baltimore to meet at that city in 1848. Several resolutions on various subjects were submitted, which were ordered to lie on the table, except those inviting to a seat in the convention, without the privilege of voting, Prof. Thomas Spencer, of Geneva, N. Y., and Prof. Robert Hare, of Philadelphia, both gentlemen being accidently present.

The report of the committee on an uniform system of nomenclature of diseases was heard, received, and ordered to be printed for the use of the members.

Two reports, by different portions of the committee, on the subject of separating the business of licensing and teaching, and committing the former to central boards of examiners in each state, were presented; one by Prof. M'Naughton, of N. Y., the other by Dr. Isaac Parrish, of Philadelphia. They did not differ materially in the views or resolutions embraced, and were both ordered to be printed for the use of the members.

The convention then proceeded to consider the report and resolutions on prelimenary education, which, after the suggestion of many amendments, and considerable discussion, were adopted with the change of a single word only.

The convention at this stage of its proceedings adopting a resolution permitting members to speak but twice on the same subject, and to occupy not more than ten minutes at any one time without consent of the convention. Adjourned at 12½, P. M., to meet again at 5 P. M.

Convention met at 5 P. M., and proceeded to take into consideration the resolutions reported by the committee on requirments for a degree. The most important feature of these resolutions was the extension of the lecture sessions of medical schools to six months. This occasioned lengthened discussion, but was finally adopted. The other resolutions were adopted with a slight amendment to one of them, and an additional one supplied, enjoining the faculties of medical schools to take some effective measures to enforce regular attendance upon lectures. The discussions upon these resolutions occupied the whole afternoon session, and the convention adjourned to meet at 9 A. M. to-morrow.

We visited this afternoon the Pennsylvania Hospital for the Insane, located two miles west of the city. This is a branch of the Pennsylvania Hospital. It has been in operation since 1840, having been from the commencement under the superintendence of Dr. Thos. S. Kirkbride. The Hospital is finely located, having extensive and comfortable accommodations, with finely cultivated grounds. Every thing, within and without, denotes system, cleanliness, comfort and taste. There are now at the institution 192 patients, forty of whom, only, are paupers. The charges are three dollars for patients from the state of Pennsylvania, and five dollars from other states. A large proportion are from the Southern States.

On our return route we stopped for a few moments at the Alms House, Blockley, styled, not inappropriately, the *pauper palace*. It is a huge establishment, consisting of several buildings enclosing a hollow square, and covering a very large area. It contains fifteen hundred inmates, about four hundred of whom are on the sick list. We did not remain sufficiently long to examine the establishment throughout, but the impression which I derived, was, that it is alltogether too extensive for the complete accomplishment of its objects, and that as regards ventilation, and comfortable accommodations for its inmates, the arrangements are by no means entirely satisfactory. I was suprised to learn that medical students have been of late excluded from this great field of instruction in clinical medicine.

*Friday 7th.*—Convention assembled at 9 A. M. A resolution was adopted inviting the venerable Dr. Coxe to take a seat in the convention. Dr. Coxe shortly entered the hall, was received with marked respect, and seated with the vice presidents.

An amendment to the report on the requirements for degrees was adopted, striking out a passage requiring each course to embrace one hundred lectures.

Several resolutions on various subjects were presented by different members, but ordered to lie on the table, the convention being disposed to proceed with the regular business, viz: the consideration of the reports of the committees.

The report on the organization of a national association was taken up.

Dr. Isaac Hays submitted an amendment providing that the association should consist of all members of the medical profession in good standing throughout the country, instead of being limited to appointed delegates. The object of the amendment was to secure the advantages of a distinct line of demarkation between regular and irregular practitioners. This proposition called out an extended discussion, but the plan of the committee was sustained by a decisive vote of the convention. The report was then adopted.

The report on medical ethics, after some discussion upon several proposed amendments, was adopted, with a few alterations of phraseology.

The report on registration of births, marriages, and deaths was adopted without discussion.

A resolution approving of the objects of the Sydenham Society was adopted.

The report on the Nomenclature of diseases being called up, was referred to the committee on Medical Education to be appointed under the new organization.

The subject of the separation of licensing and teaching now came up, and remarks were made by several members supporting various amendments and resolutions. The subject was, at length, disposed of, by the adoption of a resolution referring the two reports made to the convention to the committee on Medical Education, with instruction to report at the next annual meeting of the American Medical association. The convention now adjourned to 5½ P. M.

At the evening session, a variety of resolutions on various topics were presented, most of which were laid on the table.

A resolution was passed returning thanks to the officers and directors of the various institutions from whom invitations had been received; to the committee of arrangements on behalf of the Philadelphia delegation, and to the medical profession of the city generally for their kindness and hospitality.

A resolution of thanks to the officers of the convention was passed, also a resolution recommending the medical profession to unite in endeavoring to procure laws legalising, and providing for the prosecution of dissections in States where such laws do not exist.

After resolving that all unfinished business be referred to the American Medical Association about to be organized, it was moved and carried, that the convention resolve itself into the *American Medical Association*, and that the officers of the convention continue to act as officers of the association until others are elected.

A committee consisting of one member from each state represented, was appointed to nominate officers of the association for the ensuing year. After a short recess, the following names were submitted by the committee:—

*President.*—Dr. N. Chapman, of Pennsylvania.

*Vice Presidents.*—Dr. Knight, of Connecticut; Dr. A. H. Stevens, of New-York; Dr. Buchanan, of Tennesee; Dr. Moultrie, of South Carolina.

*Secretaries.*—Dr. Stille, of Pennsylvania; Dr. Dunbar, of Maryland; Dr. F. C. Stewart, of New-York.

*Treasurer.*—Dr. Isaac Hays, of Pennsylvania.

The foregoing ticket was unanimously elected by ballot. The officers on being apprized of their election by a committee appointed for that purpose, took their seats, and the venerable President elect returned his acknowledgements of the honor conferred on him in a few happy and impressive remarks.

On motion it was resolved to accept the invitation of the Baltimore delegation to hold the next annual meeting of the American Medical Association in that city, after which the association adj.

I have intended in this letter to give a brief synopsis of the more important of the proceedings of the convention. Some resolutions which were adopted by the convention, I find, on reviewal, I have omitted: these I will take occasion to present to our readers when the published proceedings appear. The publication of two thousand copies of the proceedings was ordered, and the expense of publication, together with other expenses, provided for by an assessment of three dollars on each member of the convention.

I reserve some reflections upon the eventful occasion which has just terminated for another time and place.

Very truly, Yours,

F. H. H. EDITOR.

*Editor ad interim.*

